# Effect of Newer Intraorifice Barriers on the Fracture Resistance of Endodontically Treated Teeth: An In Vitro Study

**DOI:** 10.7759/cureus.69463

**Published:** 2024-09-15

**Authors:** Johanna M D'souza, Ida de Noronha de Ataide, Rajan Lambor

**Affiliations:** 1 Conservative Dentistry and Endodontics, Goa Dental College and Hospital, Panaji, IND

**Keywords:** biodentine, flowable nanohybrid composite, fracture resistance, intraorifice barrier, resin-modified glasss ionomer

## Abstract

Aim: This study aimed to assess and compare the fracture resistance of endodontically treated teeth using three new intraorifice barrier materials.

Materials and methods: A total of 60 extracted human mandibular premolars having single roots were decoronated to 14 mm length, prepared up to rotary F3 ProTaper Gold files, and sealed with gutta-percha and AH Plus sealer. Specimens were divided into one control and three experimental groups (*n *= 15): Group 1, control; Group 2, Biodentine (Septodont, Saint Maur des Fosses, France); Group 3, resin-modified glass ionomer cement (RMGIC, GC Gold Label 2 LC, GC Corporation, Tokyo, Japan); and Group 4, flowable nanohybrid composite (G-aenial Universal Flo, GC Corporation, Tokyo, Japan). A 3 mm coronal gutta-percha was replaced with respective intraorifice barrier materials in the experimental groups, and the fracture resistance of all the groups was tested using the universal testing machine.

Statistical analysis: One-way analysis of variance and Tukey's post-hoc test were conducted.

Results: The experimental groups showed higher mean load values than the control group. The flowable composite showed the highest mean loads followed by Biodentine and RMGIC. The mean fracture resistance of flowable nanohybrid composite and Biodentine was significantly higher than that of the control. No statistically significant difference was observed among the other groups.

Conclusion: The flowable nanohybrid composite and Biodentine significantly improved resistance to fracture of endodontically treated teeth when compared to the control.

## Introduction

The strength of endodontically treated teeth is a critical consideration in clinical practice. Several changes occurring post-endodontic therapy can alter the mechanical properties of the tooth. Vertical root fractures are described as the third leading cause of tooth loss after dental caries and periodontal disease [1]. When compared to vital teeth, endodontically treated teeth are more vulnerable to fractures ranging from a simple cusp fracture to a catastrophic root fracture ultimately requiring extraction [2]. According to numerous reports, 11%-13% of extracted root canal-treated teeth showed vertical root fractures [3,4], making it the second most prevalent reason for the loss of endodontically treated teeth [5]. Furthermore, Bender and Freedland have also stated that vertical root fractures exhibited the highest incidence in obturated teeth [6].

These fractures are multifactorial in origin owing to the lost tooth structure and stresses induced during various endodontic and restorative procedures such as access cavity preparation, instrumentation, irrigation, high compaction forces during obturation, excessive post space preparation, incorrect post-selection, and inappropriate selection of tooth abutments for prosthesis [7].

This advocates the need for reinforcement of the remaining radicular tooth structure particularly in posterior teeth that are subjected to heavy occlusal forces. For this reinforcement, it is recommended that concentration of stress at the dentin-restoration interface be reduced by using restorative materials that have compressive strength and modulus of elasticity close to that of dentin [8].

Roghanizad and Jones first proposed the use of intraorifice barriers in root canal-treated teeth for the prevention of coronal microleakage [9]. Subsequent studies evaluated its advantages in enhancing tooth resistance to fracture following endodontic treatment.

During canal preparation, the greater taper of rotary instruments increases the susceptibility of the coronal third of the teeth to fracture, and therefore, more focus should be placed on reinforcing these fragile areas [10]. Various materials have been used as intraorifice barrier materials, but their effects on improving the strength and resistance to fracture are limited in literature. Dentin replacement materials like Biodentine, resin-modified glass ionomers (RMGIs), and newer nanohybrid composites have improved physical properties.

The present study aimed to evaluate the fracture resistance of these newer materials when used as intraorifice barriers in endodontically treated teeth. The objectives were to compare different materials as well as to determine the strongest material in terms of resistance to fracture when used as intraorifice barriers in endodontically treated teeth.

## Materials and methods

A total of 60 human premolar teeth having single roots were selected. This sample size was calculated using mean and standard deviation values from a previous study by Aboobaker et al., and a pooled standard deviation was calculated to be 67.68. Thus, using G*Power 3.1 software (G*Power, Universität Düsseldorf, Germany) and Cohen’s d method with alpha = 0.05, power of 95%, and calculated effect size f = 2.05, the sample size was estimated to be 12 per group. However, assuming the fracture of specimens and other failures during the procedure, the sample size was rounded off to 15 per group. Therefore, a total sample of 60 was determined.

The samples were first visually inspected for caries, cracks, curved roots, and resorption. Furthermore, intraoral periapical radiographs were taken to confirm the presence of a single canal and eliminate teeth with resorptive defects. Additionally, the samples were stained with 2% methylene blue dye for five seconds and thoroughly washed with distilled water to check for the presence of cracks. All the final samples were then cleaned using an ultrasonic scaler handpiece (Woodpecker HW-5L) with an ultrasonic scaler tip (G1) for the removal of debris and calculus. The samples were then stored in distilled water for two weeks until use to prevent dehydration.

A digital vernier caliper was used to determine the 14 mm length from the root apex, and using a fine-point marker the identified points were marked, and the samples were decoronated along the marking with a low-speed handpiece with a diamond disc under water coolant. The final length was reconfirmed using the vernier caliper in order to maintain homogeneity.

Obturation of the root canal

Access was gained using a round diamond bur. A 10 K file was introduced into the canal until it became visible through the apical foramen and the working length was set 1 mm short of this length. Hand filling was carried out up to 20 K file following which rotary ProTaper Gold files (Dentsply Maillefer, Ballaigues, Switzerland) were used with an endo motor (Root ZX 2, J Morita) in the crown down technique at a speed of 300 rpm with light apical pressure. The Sx was used for orifice opening followed by the shaping files S1 and S2. The canals were instrumented up to the apex using F1, F2, and F3 in a sequential manner. Irrigation was carried out with 5 ml of 5.25% sodium hypochlorite after the change of each file followed by 5 ml of 17% ethylenediaminetetraacetic acid. A final rinse of 10 ml saline was used. Paper points were used to dry the canals, and the teeth were obturated using corresponding F3 gutta-percha cones and AH Plus sealer (De Trey-Dentsply, Konstanz, Germany). Excess gutta-percha was seared using a gutta-percha cutter and burnished up to the orifice. A temporary restoration was placed, and the samples were stored in 100% humidity for eight hours at 37°C to ensure complete sealer setting.

Placement of Intraorifice Barrier

**Figure 1:**
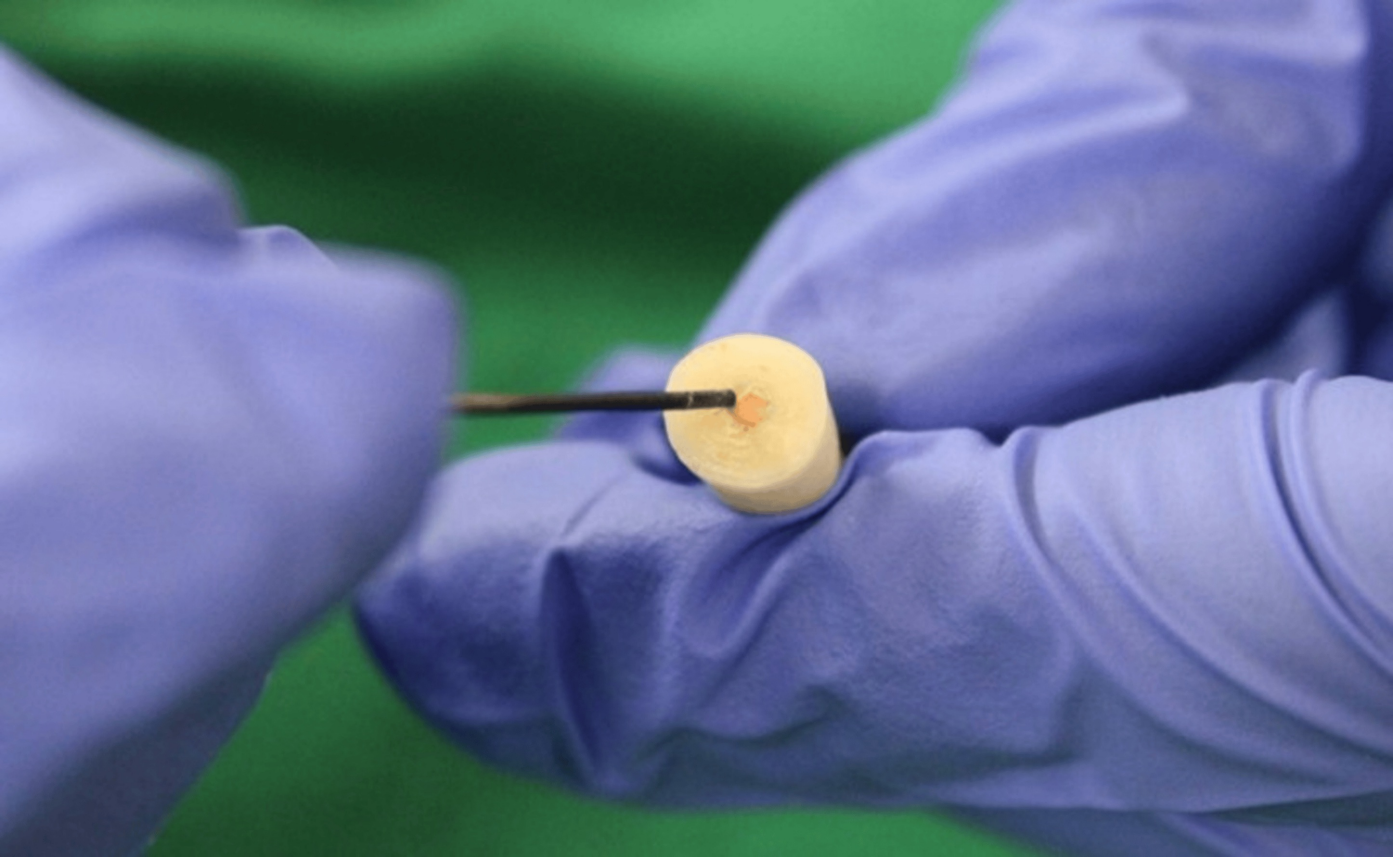
Removal of 3 mm gutta-percha using a heated plugger

Specimens were assigned to one control and three experimental groups, each consisting of 15 samples. After removal of the temporary filling material, a heated finger plugger (Dentsply Maillefer, Ballaigues, Switzerland) was carried to the specimen to sear off 3 mm of coronal gutta-percha in all experimental groups (Figure 1).

**Figure 2:**
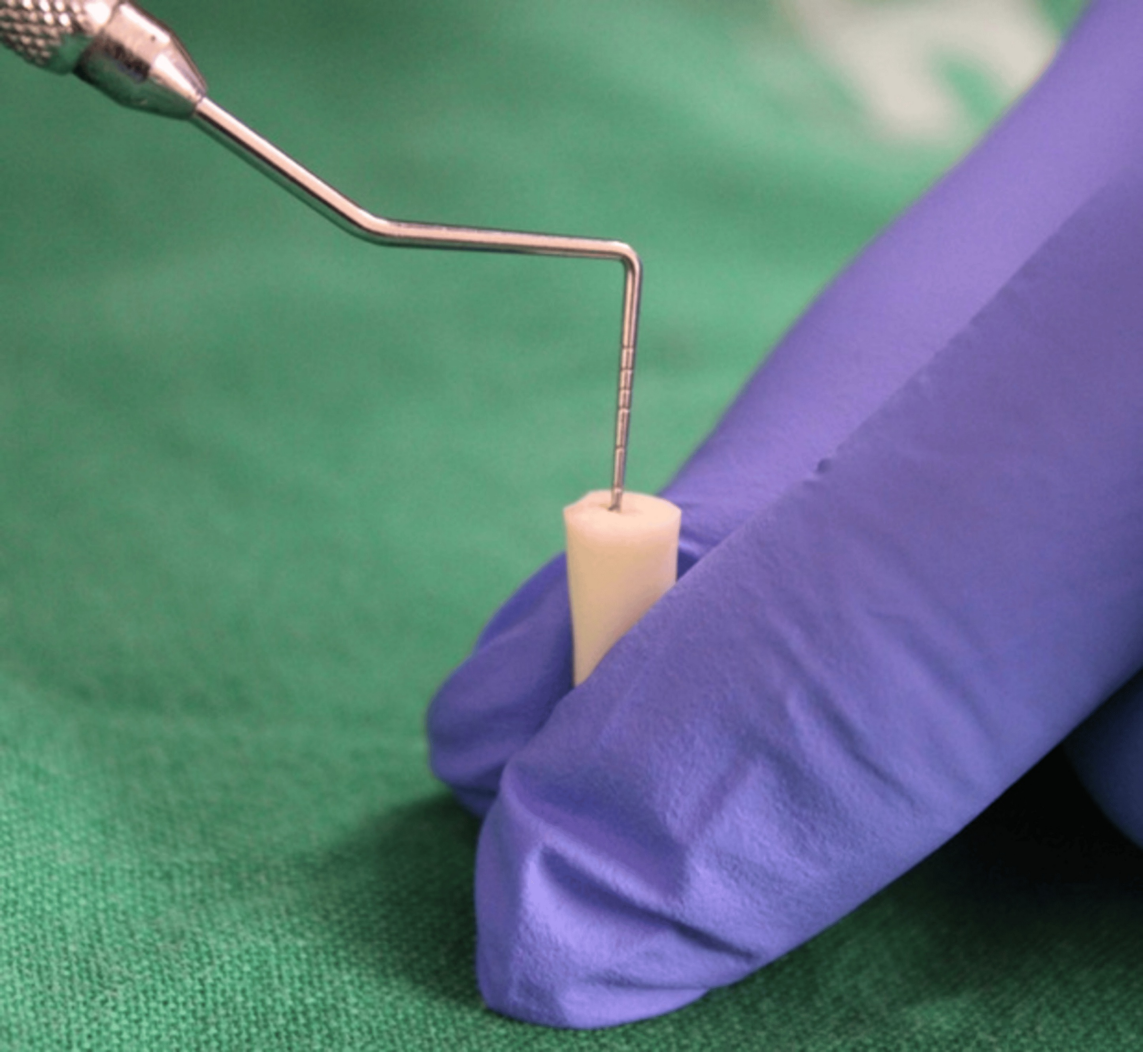
A 3 mm depth confirmed using a periodontal probe

This depth was confirmed using a periodontal probe (Figure 2). A cotton pellet moistened with 70% ethanol was utilized to remove remnants of sealer or gutta-percha.

Group 1, control group (n = 15): No intraorifice barrier was placed, and the gutta-percha was left untouched.

Group 2, Biodentine (Septodont, Saint Maur des Fosses, France) (n = 15): One capsule of powder and five drops of liquid were triturated in an amalgamator for 30 seconds according to the manufacturer's recommendation. The mix was then carried to the prepared space using an amalgam carrier and condensed with the use of a hand plugger.

Group 3, resin-modified glass ionomer cement (RMGIC) (GC Gold Label 2 LC, GC Corporation, Tokyo, Japan) (n = 15): The cavity was conditioned with GC conditioner. The cement was mixed as per manufacturer's instructions and placed with a plastic filling instrument into the space in two increments of 1.5 mm each. Each increment was light cured using 470 nm visible light curing device (Elipar DeepCure, 3M ESPE) for 20 seconds.

Group 4, flowable nanohybrid composite (G-aenial Universal Flo, GC Corporation, Tokyo, Japan) (n = 15): The surface was etched with 37% phosphoric acid for 15 seconds and rinsed for 10 seconds followed by the application of an adhesive (Tetric N-Bond Universal, Ivoclar Vivadent) and light cured for 10 seconds (Elipar DeepCure, 3M ESPE). The prepared space was restored with the flowable composite in two increments of 1.5 mm each, using 470 nm visible light curing device (Elipar DeepCure, 3M ESPE) curing at a distance of 2 mm for 20 seconds.

Specimens were then stored at 37°C at 100% humidity for 48 hours to ensure complete set of the materials.

Mounting and testing of specimens

Specimens were secured vertically in self-cure acrylic blocks of dimension 2 x 2 x 2 cm, with 9 mm of coronal root exposed. Light body elastomeric impression material was placed around the tooth roots to simulate the periodontal ligament [11].

**Figure 3:**
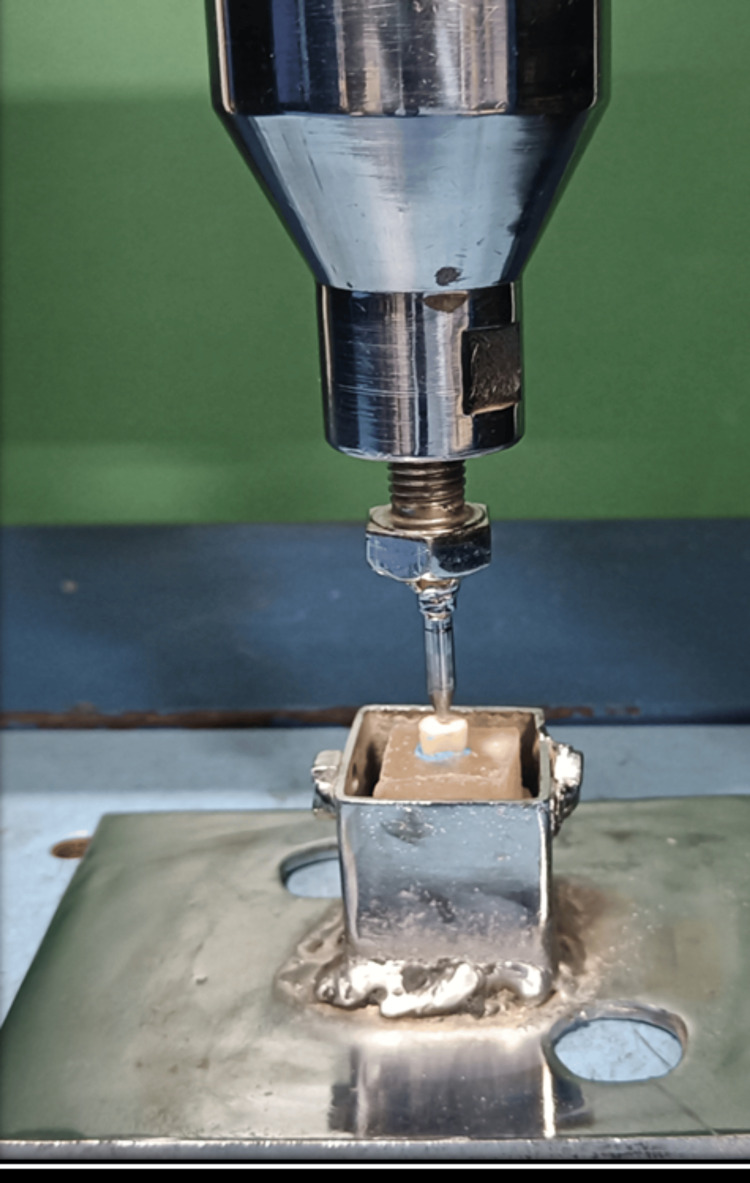
Specimen subjected to vertical force under a universal testing machine

Using a universal testing machine (Unitest 10) consisting of a 2 mm cylindrical stainless steel plunger, a compressive force at a crosshead speed of 1 mm/min was applied vertically centered over the intraorifice barrier material until fracture of the sample occurred (Figure 3). Fracture force for each specimen was quantified in newtons (N).

## Results

Statistical analysis

The IBM SPSS Statistics for Windows, Version 26 (Released 2019; IBM Corp., Armonk, New York, United States) was used to analyze the results. A comparison of the fracture resistance of the groups was made using a one-way analysis of variance test. Pairwise comparison between groups was done using Tukey's post-hoc test, and the significance level was set at 0.05. The mean and standard deviations of the four groups were calculated (Table 1). Group 4 showed the highest mean force values followed by Group 2 > Group 3 > Group 1.

**Table 1:** Mean fracture load in newtons (N) RMGIC: Resin-modified glass ionomer cement; n: sample size; N: mean fracture load in newtons; CI: confidence interval; ANOVA: analysis of variance

Groups	n	Mean (N)	Standard deviation	Shapiro-Wilk	One-way ANOVA		95% CI lower	95% CI upper
				p-value	p-value	ɳ^2^		
Control	15	186.64	36.25	0.528	0.012	0.3592	153.10	220.16
Biodentine	15	292.03	43.95	0.869			251.38	332.68
RMGIC	15	251.81	58.13	0.277			198.04	305.57
Flowable composite	15	308.03	107.93	0.265			208.21	407.84

Post-hoc Tukey's test (Table 2) indicated that the mean fracture resistance of flowable nanohybrid composite and biodentine were higher than that of the control group with a p-value of 0.013 and 0.035, respectively. Although the flowable nanohybrid composite group showed better resistance to fracture than Biodentine, this difference was not statistically significant. No statistically significant difference was found between the remaining groups.

**Table 2:** p-values of post-hoc Tukey's test for pairwise comparison RMGIC: Resin-modified glass ionomer cement; CI: confidence interval

Paired comparison of groups	Mean difference	p-value	Cohen’s d	95% CI lower	95% CI upper
Biodentine vs. control	105.39	0.035	1.5593	0.3623	2.7563
Biodentine vs. flowable composite	-16.0	0.970	-0.2367	-1.3421	0.8688
Biodentine vs. RMGIC	40.22	0.685	0.5951	-0.5223	1.7124
Control vs. flowable composite	-121.39	0.013	-1.7960	-3.0220	-0.5699
Control vs. RMGIC	-65.17	0.296	-0.9642	-2.1042	-0.1758
Flowable composite vs. RMGIC	56.22	0.421	0.8318	-0.2989	1.9624

## Discussion

As endodontically treated teeth are more prone to fracture, this study assessed the efficacy of different intraorifice materials in improving the fracture resistance of teeth that have undergone endodontic treatment. The experimental groups demonstrated superior fracture resistance in comparison to the control group. Thus, placing an additional barrier 3 mm into the root canal could improve the resistance to fracture. Numerous studies have stated that at this depth, the barrier material had better sealing which could also prevent reinfection as opposed to when placed at 1, 2, or 4 mm [9,12,13]. The presence of a barrier material also improved the coronal seal which was shown to significantly reduce the signs of apical periodontitis in dogs [14].

The quantity of remaining tooth structure determines the strength of the tooth [15]. The pericervical dentin, located near the alveolar crest extending about 4 mm coronally and apically from the crestal bone, is vital for stress transfer along the length of the tooth. The loss of this pericervical dentin can increase the susceptibility of the tooth to fracture [8]. Furthermore, the aggressive use of greater taper rotary instruments weakens the radicular dentine [16]. Rundquist et al. [10] stated that in an obturated tooth, the occlusal forces generate maximum stresses at the cervical portion of the root which increases as the taper of the instrument increases. Therefore, to reinforce such teeth, the concentration of stress at the dentin-restoration interface should be reduced by using materials having a modulus of elasticity comparable to that of dentin, which is about 14-16 GPa [17].

Additionally, as gutta-percha does not bond to the radicular dentin, emphasis is placed on using materials that adhere to the root dentine and reinforce the remaining tooth structure [18]. Various experiments have included intraorifice barriers consisting of bioceramics, resin-based composites, glass-ionomer cements [8,9,12], etc. The materials selected for this study were based on their ability to adhere to the tooth structure, having low elastic modulus, and those which did not affect the adhesion of the final restoration.

In the experimental groups, 3 mm of barrier material replaced the gutta-percha to compensate for the lost pericervical dentin, which improved fracture resistance as the restorative materials flexed under occlusal loading evenly distributing the stresses along the dentin-restoration interface [8,19]. The mean values also revealed that fracture resistance of the roots was influenced by the type of barrier placed as stated by Nagas et al. [19].

In the present study, flowable nanohybrid composite (G-ænial Universal Flo) exhibited higher fracture resistance than Biodentine even though the mean difference was not statistically significant. These results were contradictory to a previous study [8] that reported highest values with Biodentine (modulus of elasticity 22 GPa). This could be due to the low viscosity of the resin that allowed better adaptation of the flowable to the internal dentin walls [20]. Flowable nanohybrid composite resin (modulus of elasticty 4.7-7.6 GPa) comprises of nanosized filler particles (200 nm) and a filler load of 69% as opposed to 20%-25% in conventional flowable resins which imparts high flexural strength and reduced polymerization shrinkage [21]. A study conducted by Yasa et al. [11] reported similar results where composite performed better than Biodentine.

However, several studies have demonstrated that bioceramics exhibit superior performance compared to composite resins due to the lack of polymerization shrinkage and hygroscopic expansion [22]. Biodentine due to its improved handling, faster set, high compressive strength, and better sealing ability was selected over mineral trioxide aggregate (MTA) for the present study. The smaller particle size facilitates calcium and silicon ions to penetrate deeper into dentinal tubules forming tags that create micromechanical adhesion to the tooth. Its compressive strength is comparable to that of dentin which enables even distribution of stresses along the interface of the tooth and restoration during occlusal loading [23].

RMGIC also showed higher mean value than the control group. This resin-reinforced material is easy to manipulate and exhibits high flexural strength and modulus of elasticity (10-14 GPa) similar to that of dentin. However, among the experimental groups, it exhibited the lowest mean values which could be attributed to the increased polymerization shrinkage and low rigidity of the material when compared to resin-based composites [8]. Nevertheless, Nagas et al. [19] and Aboobaker et al. [24] have reported both RMGIC and flowable resin to be equally effective as intraorifice barriers. The lowest fracture resistance was noted in the control group which was in accordance with numerous other studies [8,17,19].

All efforts for standardization of the study were made: The specimens included only mandibular premolar teeth randomly allocated to the groups, having similar dimensions and root lengths standardized to 14 mm. Canal preparation and obturation was done using the same technique. Moreover, periodontal ligament was simulated. Additionally, only straight rooted teeth were selected as a curved root would have modified the stress distributions within the tooth and obscured the results. Forces were applied along the long axis of the tooth which allowed uniform transmission to the specimens [10,15,19]. 

However, the present study evaluated fracture resistance by load application in a single direction and at a single point which does not simulate oral conditions. Moreover, the possibility of using other restorative materials for this purpose needs to be explored. Hence, within these limitations, the results of this in vitro study can be applied to a clinical scenario with caution as further studies may be necessary.

## Conclusions

The lowest mean fracture resistance was noted in the control group which indicates its reduced ability to withstand occlusal forces warranting the need for intraorifice barriers to strengthen endodontically treated teeth. Among the tested materials, flowable nanohybrid composite exhibited maximum resistance to fracture followed by Biodentine and RMGIC. Hence, from the results of the present study, it can be concluded that intraorifice barriers can be considered as a crucial choice in reducing post-endodontic root fractures.
